# A Large Cohort Study Reveals the Association of Elevated Peripheral Blood Lymphocyte-to-Monocyte Ratio with Favorable Prognosis in Nasopharyngeal Carcinoma

**DOI:** 10.1371/journal.pone.0083069

**Published:** 2013-12-27

**Authors:** Jing Li, Rou Jiang, Wen-Sheng Liu, Qing Liu, Miao Xu, Qi-Sheng Feng, Li-Zhen Chen, Jin-Xin Bei, Ming-Yuan Chen, Yi-Xin Zeng

**Affiliations:** 1 Collaborative Innovation Center for Cancer Medicine, State Key Laboratory of Oncology in South China, Sun Yat-sen University Cancer Center, Guangzhou, People's Republic of China; 2 Department of Experimental Research, Sun Yat-sen University Cancer Center, Guangzhou, People's Republic of China; 3 Department of Nasopharyngeal Carcinoma, Sun Yat-sen University Cancer Center, Guangzhou, People's Republic of China; 4 Department of Epidemiology, Sun Yat-Sen University Cancer Center, Guangzhou, People's Republic of China; Kenya Medical Research Institute (KEMRI), Kenya

## Abstract

**Background:**

Nasopharyngeal carcinoma (NPC) is an endemic neoplasm in southern China. Although NPC sufferers are sensitive to radiotherapy, 20–30% of patients finally progress with recurrence and metastases. Elevated lymphocyte-to-monocyte ratio (LMR) has been reported to be associated with favorable prognosis in some hematology malignancies, but has not been studied in NPC. The aim of this study was to evaluate whether LMR could predict the prognosis of NPC patients.

**Methods:**

A retrospective cohort of 1,547 non-metastatic NPC patients was recruited between January 2005 and June 2008. The counts for peripheral lymphocyte and monocyte were retrieved, and the LMR was calculated. Receiver operating characteristic curve analysis, univariate and multivariate COX proportional hazards analyses were applied to evaluate the associations of LMR with overall survival (OS), disease-free survival (DFS), distant metastasis-free survival (DMFS) and loco-regional recurrence-free survival (LRRFS), respectively.

**Results:**

Univariate analysis revealed that higher LMR level (≥5.220) was significantly associated with superior OS, DFS and DMFS (P values <0.001). The higher lymphocyte count (≥2.145×10^9^/L) was significantly associated with better OS (P = 0.002) and DMFS (P = 0.031), respectively, while the lower monocyte count (<0.475×10^9^/L) was associated with better OS (P = 0.012), DFS (P = 0.011) and DMFS (P = 0.003), respectively. Multivariate Cox proportional hazard analysis showed that higher LMR level was a significantly independent predictor for superior OS (hazard ratio or HR  = 0.558, 95% confidence interval or 95% CI  = 0.417–0.748; P<0.001), DFS (HR  = 0.669, 95% CI  = 0.535–0.838; P<0.001) and DMFS (HR = 0.543, 95% CI  = 0.403–0.732; P<0.001), respectively. The advanced T and N stages were also independent indicators for worse OS, DFS, and DMFS, except that T stage showed borderline statistical significance for DFS (P = 0.053) and DMFS (P = 0.080).

**Conclusions:**

The elevated pretreatment peripheral LMR level was a significant favorable factor for NPC prognosis and this easily accessed variable may serve as a potent marker to predict the outcomes of NPC patients.

## Introduction

Nasopharyngeal carcinoma (NPC) is a squamous-cell carcinoma that arises in the upper lining epithelium of the nasopharynx [1]. The neoplasm exhibits a remarkable geographic distribution, which is prevalent in southern China, South-East Asia, North Africa, the Arctic nations of Alaska and Greenland. The annual incidence of NPC reaches about 25 per 100,000 individuals in the most prevalent regions, which is 25-fold higher than that in the western world [2]. This malignancy is radiosensitive and radiotherapy is the standard treatment for NPC. Although the overall 5-year survival rate of NPC patients is around 80%, 20–30% patients develop distant metastasis and/or loco-regional recurrence [3]. Therefore, a valuable marker to predict outcomes or prognosis for NPC patients is desirable to facilitate individualized treatments and thus better outcomes for NPC patients.

Inflammation has long been associated with the development of cancers, and chronic systemic inflammatory response has been clearly implicated in the progressive process and subsequent poor outcomes of cancer patients [4]. Lymphocytes and monocytes are key immune cells in the inflammatory response, and have been independently associated with the prognosis of various malignancies, such as gastric cancer [5], acute lymphoblastic leukemia [6], lymphoma [7], hepatocellular carcinoma [8] and NPC [9], [10]. Interestingly, the pretreatment lymphocyte-to-monocyte ratio (LMR) was reported as a prognostic factor for clinical outcomes in diffuse large-B-cell lymphoma and Hodgkin's lymphoma [7], [9].

Studies have reported that NPC and lymphoma shared similar genetic susceptibility [11], and are also partially EBV related [12], [13], suggesting common mechanisms in the etiologies between the two types of malignancy, therefore, we hypothesized that lymphocytes, monocytes and LMR may also play important role in NPC. Here, we carried out a large-scale retrospective cohort study on NPC, attempting to investigate the prognostic value of LMR for the disease. To our knowledge, this is the first large-scale study on the association of LMR and NPC.

## Materials and Methods

### Patients' recruitment and data collection

All 1,575 participants were histologically diagnosed as non-metastatic NPC and subsequently treated at Sun Yat-sen University Cancer Center (SYSUCC) between January 2005 and June 2008. Before therapeutic regimens were proposed, pretreatment evaluations were conducted for each patient, including physical and neurologic examinations, hematology and biochemistry profiling, contrast-enhanced computed tomography (CT scan) or magnetic resonance imaging (MRI) of the head and neck, chest radiography, abdominal ultrasonography, emission CT or positron emission tomography (PET). All patients were staged according to the sixth edition of the UICC/AJCC TNM classification system. This study was approved by the ethics committees of SYSUCC. All patients wrote informed consent documents prior to participating in this study.

As part of the physical examinations, peripheral blood was collected before treatment, and both peripheral lymphocytes and monocytes were counted by using the automated hematology analyzer Sysmex XE-5000 (Sysmex, Kobe, Japan). The peripheral LMR was calculated as the ratio of absolute counts between peripheral lymphocyte and monocyte. All patients had no self-reported acute infection and hematologic disorders, indicating that the cell counts could represent the normal baseline value. Finally, we excluded 6 patients with missing pretreatment lymphocyte count and monocyte count and 28 patients unable to be followed up; so, 1,547 patients were remained for further analyses.

### Treatment and follow-up

Radiotherapy with or without chemotherapy remains the standard care for NPC [14]. All patients were treated with standard curative radical radiotherapy, including 2-division conventional radiotherapy (2D-CRT) or intensity-modulated radiotherapy as described previously [14]. Briefly, all target volumes were outlined slice by slice in the treatment planning system based on enhanced CT scans. The radiation dose was 60∼72 Gy at the nasopharyngeal region and 50∼66 Gy at the regional lymph nodes. The majority of patients at stage III or IV (78.4%; 1,213 out of 1,547) and minority of patients at stage II (16.0%; 247 out of 1,547) were treated with a platinum-based chemotherapy. When required, salvage treatments (including surgery, branchy therapy, and chemotherapy) were provided in the event of documented disease recurrence or persistence.

The patients were followed up every three months in the first three years, and every six months thereafter or until death. The last follow-up date was May 30, 2013 for all the available patients. Local recurrence was established by fiberoptic endoscopy, MRI and biopsy. Distant metastases were diagnosed based on clinical symptoms, physical examination, and imaging methods including CT-scan, bone scan, and abdominal sonography or PET-CT.

### Statistical analysis

Receiver operating characteristic (ROC) curve analysis was performed to select the most appropriate cut-off points for the counts of lymphocyte and monocyte as well as LMR to stratify patients at a high risk of malignancy-related death. The score at the point with both maximum sensitivity and specificity was selected as the best cut-off value. Survival outcomes were dichotomized by survival (alive versus death) and relapse (local failure and/or distant metastasis versus no local failure and/or distant metastasis) in the ROC analysis. The following endpoints were assessed: overall survival (OS), disease-free survival (DFS), distant metastasis-free survival (DMFS) and loco-regional recurrence-free survival (LRRFS). OS was defined as the duration from diagnosis until the date of death from any causes, or date of the last follow-up. The event for DFS was the duration between the date of being diagnosed and the date of having events of loco-regional recurrence and/or metastasis, or date of the last follow-up. DMFS was defined as the duration from diagnosis until the date of metastasis, or date of the last follow-up. The event for LRRFS was the duration between the date of being diagnosed and the date of having event of loco-regional recurrence or date of the last follow-up. These endpoints were analyzed and compared by using the Kaplan-Meier method and the log-rank test. Categorical variables were compared using χ^2^ tests. Continuous variables, reported as median age, were compared using the Wilcoxon rank-sum test. Multivariate analyses with the Cox proportional hazards model were used to test independence, significance, and hazard discrimination, respectively. Covariates included in the model are given in the result tables as previously reported manner [15]. A two-tailed P value <0.05 was considered statistically significant. The correlations between lymphocyte or monocyte counts and their ratio were evaluated, respectively, by spearman's rank correlation coefficient. Above analyses were done by using SPSS software (version 16.0, SPSS Inc, Chicago, USA).

## Results

### Patients' characteristics

The patients' characteristics were summarized in Table 1. As shown, the median age was 51 years (range: 6–87 years), and the female ratio was 27.3%. 1213 patients (78.4%) were diagnosed at late stages (III and IV), and the other 334 patients (21.6%) were at early stages (I and II), respectively. The mean counts of lymphocyte and monocyte were 2.13×10^9^/L (range: 0.2–5.4×10^9^/L) and 0.46×10^9^/L (range: 0.1–4.4×10^9^/L), respectively. The mean LMR level was 5.48 (range: 0.4–32). The median follow-up duration was 67.07 months (from 1.41 to 99.02 months). By the last follow-up, 149 patients (9.6%) developed loco-regional recurrences, 207 patients (13.4%) developed distant metastases, and 17 patients (1.1%) developed both distant metastases and loco-regional recurrences. The 5-year OS, DFS, DMFS and LRRFS, were 86.1%, 78.1%, 86.6% and 90.5%, respectively. Among the 215 deaths, 211 patients died of NPC with recurrences or metastases, one died of cardiovascular disease, one died of suicide, and the others two died of radiation encephalopathy.

**Table 1:** Baseline clinical characteristics of the 1547 nasopharyngeal carcinoma patients according to lymphocyte-to-monocyte ratio.

characteristic	Overall	LMR<5.22 (n = 866)	LMR≥5.22 (n = 681)	p-value
**Median age (years)**	51	51	51	0.406^a^
**Gender (n, % female)**	423/1547 (27.3%)	212 (24.5%)	211 (31.0%)	0.004^b^
**T-classification**				
T1–T2	534	273	261	0.005^b^
T3–T4	1013	593	420	
**N-classification**				
N0–N1	846	465	381	0.377^b^
N2–N3	701	401	300	
**Overall stage**				
I–II	334	163	171	0.003^b^
III–IV	1213	703	510	
**Treatmen**t				
RT	493	273	220	0.743^b^
CRT	1054	593	461	
**Lymphocyte Count (10^9^/L)**	2.13 (0.2–5.4)*	1.96 (0.2–4.4)*	2.34 (1–5.4)*	<0.001^a^
**Monocyte Count (10^9^/L)**	0.46 (0.1–4.4)*	0.57 (0.2–4.4)*	0.32 (0.1–0.8)*	<0.001^ a^

Representing mean and range in the bracket.

The mean LMR level was 5.48 (ranges: 0.4–32).

Abbreviation: CRT  = chemoradiotherapy; RT  =  radiotherapy; LMR  =  lymphocyte-to-monocyte ratio.

aWilcoxon rank-sum test.

bχ2 test by two-sided Pearson's exact test.

The cut-off points of LMR, lymphocyte count and monocyte count for survival outcomes were determined by ROC curve analyses, which revealed that the LMR cut-off points for OS, DFS, DMFS and LRRFS were5.220, 4.536, 4.775 and 5.718, respectively (Figure S1). The LMR cut-off point of 5.220 for OS was selected as the uniform point in the survival analyses and all patients were divided into either high- (LMR ≥5.220) or low- (LMR <5.220) LMR groups. Similarly, lymphocyte count of 2.145×10^9^/L and monocyte count of 0.475×10^9^/L were selected as the optimal cut-off points for survival analyses.

The distributions of baseline LMR and other clinical characteristics were shown in Table 1 and Table S1. Patients with higher LMR level (≥5.220) had a lower incidence of advanced T and overall stages (P = 0.005 and P = 0.003, respectively). Female patients usually presented lower LMR at diagnosis (P = 0.004). The mean lymphocyte count in the overall patients, the lower LMR group and the higher LMR group was 2.13, 1.96 and 2.34 respectively (P<0.001). The mean monocyte count was 0.46, 0.57 and 0.32 respectively in the overall patients, the lower LMR group and the higher LMR group (P<0.001) (Table 1).

### Univariate analysis of LMR as a prognostic factor for OS, DFS, DMFS and LRRFS

**Figure 1:**
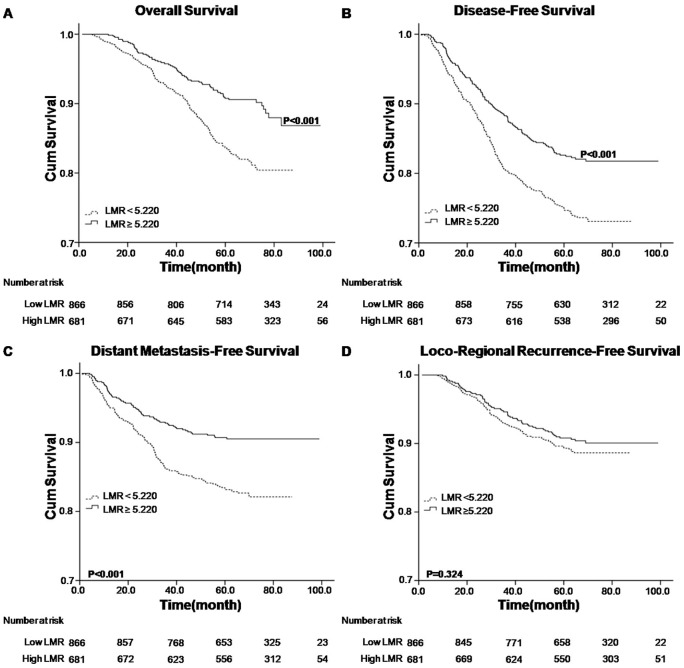
Kaplan–Meier survival analysis of baseline LMR in NPC patients. A. OS curves for LMR; B. DFS curves for LMR; C. DMFS curves for LMR; D. LRRFS curves for LMR. LMR, lymphocyte-to-monocyte ratio; NPC, nasopharyngeal carcinoma; OS, overall survival; DFS, disease-free survival; DMFS, distant metastasis-free survival; LRRFS, loco-regional recurrence-free survival.

**Figure 2:**
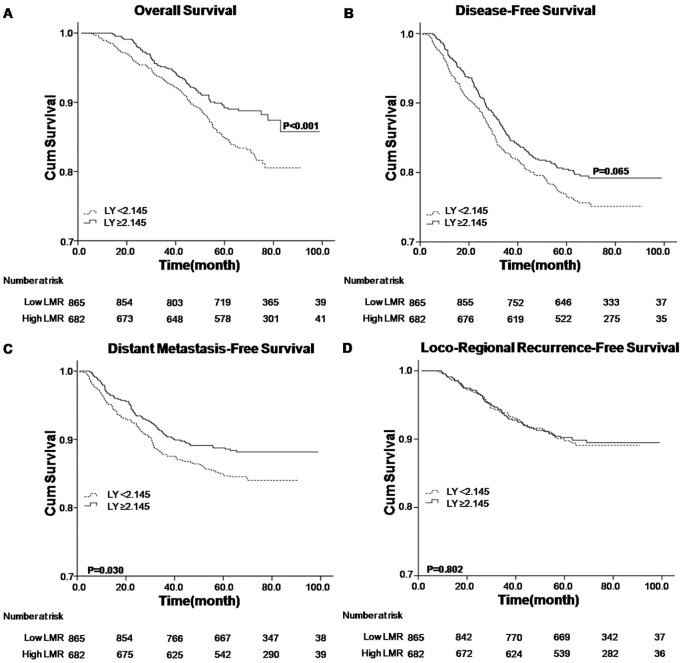
Kaplan–Meier survival analysis of baseline lymphocyte count (LY) in NPC patients. A. OS curves for LY; B. DFS curves for LY; C. DMFS curves for LY; D. LRRFS curves for LY. LY, lymphocyte count; NPC, nasopharyngeal carcinoma; OS, overall survival; DFS, disease-free survival; DMFS, distant metastasis-free survival; LRRFS, loco-regional recurrence-free survival.

**Figure 3:**
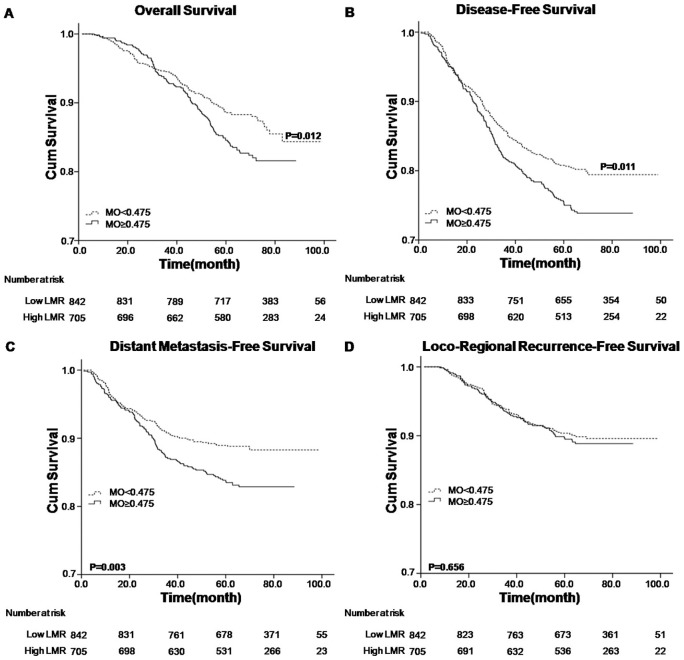
Kaplan–Meier survival analysis of baseline monocyte count (MO) in NPC patients. A. OS curves for MO; B. DFS curves for MO; C. DMFS curves for MO; D. LRRFS curves for MO. MO, monocyte count; NPC, nasopharyngeal carcinoma; OS, overall survival; DFS, disease-free survival; DMFS, distant metastasis-free survival; LRRFS, loco-regional recurrence-free survival.

Analyzed factors included gender, age, T stage, N stage, overall stage, treatment modality, lymphocyte count, monocyte count and LMR status. Univariate analysis revealed that higher LMR level (≥5.220) was associated with superior OS, DFS and DMFS (P value <0.001) (Table 2 and Figure 1A, 1B and 1C). The higher lymphocyte count (≥2.145×10^9^/L) was associated with better OS (P = 0.002) and DMFS (P = 0.031), respectively (Table 2 and Figure 2A and 2C), while the lower monocyte count (<0.475×10^9^/L) was associated with better OS (P = 0.012), DFS (P = 0.011) and DMFS (P = 0.003), respectively (Table 2 and Figure 3A, 3B and 3C). Other variables including female, younger age, early T, N and overall stage, radiotherapy were considered favorable factors for OS, DFS and DMFS (Table 2). None of the factors were associated with LRRFS, except that male was seemed to be associated with inferior LRRFS (P = 0.025).

**Table 2:** Univariate analysis of prognostic factors for patients with nasopharyngeal carcinoma.

Factor	Number (%)	5-year OS				5-year DFS					5-year DMFS			5-year LRRFS			
		Deaths (%)	Overall observation time (months)	HR (95% CI)	*P* value	Progression (%)	Overall observation time (months)	HR (95% CI)	*P* value	Metastasis (%)	Overall observation time (months)	HR (95%CI)	*P* value	Recurrenve (%)	Overall observation time (months)	HR (95% CI)	*P* value
**Gender**																	
Female	423 (27.3%)	35 (8.3%)	40.69±17.68	1 (r0065ference)		64 (15.1%)	28.59±15.78	1 (reference)	<0.001	37 (8.7%)	26.62±18.45	1 (reference)	0.001	30 (7.1%)	31.31±10.35	1 (reference)	0.025
Male	1124 (72.7%)	180 (16.0%)	39.59±17.16	2.056 (1.431, 2.953)	<0.001	275 (24.5%)	27.91±16.41	1.739 (1.325, 2.283)		170 (15.1%)	24.56±15.40	1.831 (1.283, 2.613)		119 (10.6%)	32.53±16.37	1.579 (1.058, 2.356)	
**Age (years**)																	
<51	729 (47.1%)	81 (10.3%)	39.1±16.0	1 (reference)	<0.001	148 (20.3%)	27.39±16.13	1 (reference)	0.148	82 (11.2%)	23.45±15.50	1 (reference)	0.02	72 (9.9%)	31.43±15.48	1 (reference)	0.879
≥51	818 (52.9%)	134 (17.7%)	40.09±17.81	1.926 (1.448, 2.561)		191 (23.3%)	28.55±16.40	1.171 (0.945, 1.452)		125 (15.3%)	25.90±16.24	1.393 (1.054, 1.841)		77 (9.4%)	33.08±15.24	0.975 (0.707, 1.345)	
**T status**																	
T1-T2	534 (34.5%)	42 (14.6%)	33.61±18.89	1 (reference)	<0.001	90 (16.9%)	29.04±17.61	1 (reference)	<0.001	47 (8.8%)	22.83±16.84	1 (reference)	<0.001	47 (8.8%)	35.15±15.72	1 (reference)	0.32
T3-T4	1013 (65.5%)	173 (19.1%)	41.26±16.49	2.301 (1.642, 3.224)		249 (24.6%)	27.68±15.78	1.546 (1.215, 1.968)		160 (15.8%)	25.55±15.69	1.880 (1.358, 2.602)		102 (10.1%)	30.97±15.04	1.192 (0.844, 1.684)	
**N status**																	
N0-N1	846 (54.7%)	93 (11.0%)	42.60±16.43	1 (reference)	<0.001	158 (18.7%)	31.10±16.98	1 (reference)	<0.001	84 (9.9%)	28.95±17.14	1 (reference)	<0.001	81 (9.6%)	33.09±16.60	1 (reference)	0.696
N2-N3	701 (45.3%)	122 (17.4%)	37.61±17.54	1.679 (1.282, 2.199)		181 (25.8%)	25.37±15.17	1.471 (1.188, 1.821)		123 (17.5%)	22.18±14.54	1.867 (1.414, 2.463)		68 (9.7%)	31.33±13.72	1.066 (0.772, 1.472)	
**Overall stage**																	
I-II	334 (21.6%)	18 (5.4%)	29.75±18.21	1 (reference)	<0.001	49 (14.7%)	30.47±19.03	1 (reference)	<0.001	21 (6.3%)	21.90±18.05	1 (reference)	<0.001	29 (8.7%)	36.03±17.70	1 (reference)	0.374
III-IV	1213 (78.4%)	197 (16.2%)	40.68±16.87	3.245 (2.003, 5.259)		290 (23.9%)	27.63±15.76	1.749 (1.292, 2.367)		186 (15.3%)	25.27±15.72	2.585 (1.646, 4.059)		120 (9.9%)	31.38±14.64	1.202 (0.801, 1.803)	
**Treatment**																	
RT	493 (31.9%)	50 (10.1%)	41.9±16.48	1 (reference)	0.003	86 (17.4%)	30.34±16.86	1 (reference)	0.003	44 (8.9%)	29.9±18.13	1 (reference)	<0.001	47 (9.5%)	30.47±15.16	1 (reference)	0.779
CRT	1054 (68.1%)	165 (15.7%)	31.92±17.42	1.625 (1.184, 2.229)		253 (24.0%)	27.26±16.02	1.450 (1.135, 1.851)		163 (15.5%)	23.59±15.10	1.825 (1.308, 2.546)		102 (9.7%)	33.12±15.41	1.051 (0.744, 1.484)	
**Lymphocyte Count (10^9^/L)**																	
<2.145	865 (55.9%)	141 (16.3%)	39.84±18.21	1 (reference)	0.002	204 (23.6%)	27.71±17.01	1 (reference)	0.065	130 (15.0%)	24.86±16.73	1 (reference)	0.031	84 (9.7%)	32.72±15.78	1 (reference)	0.802
≥2.145	682 (44.1%)	74 (10.9%)	39.63±15.25	0.647 (0.489, 0.858)		135 (19.8%)	28.55±15.13	0.815 (0.656, 1.013)		77 (11.3%)	25.04±14.67	0.733 (0.553, 0.972)		65 (9.5%)	31.73±14.82	0.959 (0.694, 1.326)	
**Monocyte Count (10^9^/L)**																	
<0.475	842 (54.4%)	101 (12.0%)	39.41±16.92	1 (reference)	0.012	164 (19.5%)	27.34±16.12	1 (reference)	0.011	93 (11.0%)	23.25±15.44	1 (reference)	0.003	79 (9.4%)	31.91±15.20	1 (reference)	0.656
≥0.475	705 (45.6%)	114 (16.2%)	40.59±17.96	1.409 (1.078, 1.843)		175 (24.8%)	28.70±16.43	1.318 (1.065, 1.631)		114 (16.2%)	26.30±16.30	1.504 (1.144, 1.978)		70 (9.9%)	21.71±15.56	1.076 (0.780, 1.484)	
**LMR**																	
<5.220	866 (56.0%)	149 (17.2%)	39.41±16.92	1 (reference)	<0.001	221 (25.5%)	27.61±16.47	1 (reference)	<0.001	145 (16.7%)	25.46±16.48	1 (reference)	<0.001	88 (10.2%)	31.61±15.26	1 (reference)	0.325
≥5.220	681 (44.0%)	66 (9.7%)	40.59±17.96	0.527 (0.394, 0.705)		118 (17.3%)	28.84±15.93	0.644 (0.515, 0.805)		62 (9.1%)	23.69±14.70	0.522 (0.388, 0.703)		61 (9.0%)	33.26±15.50	0.849 (0.612, 1.177)	

Abbreviation: OS  =  Overall Survival; DFS  =  Disease-Free Survival; DMFS  =  Distant Metastasis-Free Survival; LRRFS  =  Loco-Regional Recurrence-Free Survival; CRT  =  chemoradiotherapy; RT =  radiotherapy; LMR  =  lymphocyte-to-monocyte ratio; HR  =  Hazard Ratio, derived from COX proportional hazard model.

### Multivariate analysis of LMR as an independent prognostic factor for OS, DFS and DMFS

All the characteristics, such as gender, age, T stage, N stage, overall stage, treatment modality and LMR status and prognostic measures were included in the multivariate analysis, comparing the higher and the lower LMR groups (Table 3). The results showed that higher LMR level was a significantly independent predictor for the favorable prognostic measures, including OS (hazard ratio or HR  = 0.558, 95% confidence interval or 95% CI  = 0.417–0.748; P<0.001), DFS (HR  = 0.669, 95% CI  = 0.535–0.838; P<0.001) and DMFS (HR  = 0.543, 95% CI  = 0.403–0.732; P<0.001), respectively. The advanced T and N stages were also independent indicators for inferior OS, DFS, and DMFS, except that T stage showed borderline statistical significance for DFS (P = 0.053) and DMFS (P = 0.080). In addition, gender was shown as an independent factor for all these four prognostic measures and younger age was another independent factor for superior OS and DMFS. Likelihood ratio chi-square test showed that multivariate statistical model with LMR was superior to that without LMR to predict the outcomes (OS: χ^2^ = 16.253, P<0.001; DFS: χ^2^ = 12.707, P<0.001; DMFS: χ^2^ = 17.157, P<0.001; LRRFS: χ^2^ = 0.536, P = 0.464).

**Table 3:** Multivariate analysis of independent prognostic factors for patients with nasopharyngeal carcinoma (n = 1547).

Variable	OS		DFS		DMFS		LRRFS	
	HR (95% CI)	*P* value	HR (95% CI)	*P* value	HR (95% CI)	*P* value	HR (95% CI)	*P* value
Gender (male vs. female)	1.896 (1.316–2.731)	0.001	1.688 (1.283–2.219)	<0.001	1.722 (1.203–2.464)	0.003	1.588 (1.061–2.377)	0.025
Age (≥ vs. <51 years)	1.929 (1.447–2.572)	<0.001	1.178 (0.949–1.462)	0.139	1.424 (1.075–1.885)	0.014	0.953 (0.690–1.317)	0.770
T status (T3-T4 vs. T1–T2)	1.626 (1.037–2.548)	0.034	1.413 (0.996–2.006)	0.053	1.476 (0.955–2.282)	0.080	1.181 (0.686–2.031)	0.549
N status (N2–N3 vs. N0–N1)	1.419 (1.045–1.926)	0.025	1.383 (1.073–1.783)	0.012	1.632 (1.183–2.251)	0.003	1.044 (0.705–1.548)	0.829
Overall stage (III–IV vs. I–II)	1.621 (0.816–3.218)	0.168	0.991 (0.608–1.615)	0.969	1.181 (0.609–2.291)	0.623	1.047 (0.515–2.129)	0.899
Treatment (CRT vs. RT)	1.156 (0.824–1.622)	0.402	1.183 (0.906–1.545)	0.217	1.357 (0.948–1.943)	0.095	0.948 (0.647–1.389)	0.785
LMR(≥ vs.<5.22)	0.558 (0.417–0.748)	<0.001	0.669 (0.535–0.838)	<0.001	0.543 (0.403–0.732)	<0.001	0.885 (0.637–1.229)	0.466

Abbreviation: OS  =  Overall Survival; DFS  =  Disease-Free Survival; DMFS  =  Distant Metastasis-Free Survival; LRRFS  =  Loco-Regional Recurrence-Free Survival; CRT  =  chemoradiotherapy; RT  =  radiotherapy; LMR  =  lymphocyte-to-monocyte ratio; HR  =  Hazard Ratio, derived from COX proportional hazard model. In this analysis, T status, N status and tumor stage were divided into two groups. For T status: T1-2 and T3-4; for N status:N0-1 and N2-3; for tumor stage: I–II and III–IV.

In addition, both lymphocyte count and monocyte count were analyzed for their independences from the other covariates in the COX model (Table S2). LMR is not included here, considering that LMR was simply derived as the ratio between the lymphocyte and the monocyte counts and was related to lymphocyte count (correlation r = 0.380, P<0.001) or monocyte count (correlation r = −0.766, P<0.001) [16], [17]. The results showed that the lymphocyte count (≥2.145×10^9^/L) was an independent factor for favorable prognostic measures, while the monocyte count (≥0.475×10^9^/L) was an independent inferior prognostic factor for NPC patients (Table S2).

## Discussion

Accumulating studies have suggested a strong link between inflammation and cancer, where the pretreatment peripheral inflammatory cells, including neutrophils, lymphocytes and monocytes, were significantly associated with prognosis in different kinds of cancers [10], [18], [19], [20]. As part of the functional relevance, inflammatory responses lead to chronic oxidative stress and generate oxygen free radical, which has been shown with abilities to stimulate cancer initiation, promotion and progression [21], [22], [23]; moreover, an important component of inflammatory infiltrating leukocytes, tumor-associated macrophages (TAMs) may interact with tumor cells to promote tumor development by producing various cytokines and chemokines. Here, we have performed a large-scale cohort study on NPC to evaluate the prognostic values of peripheral lymphocytes and monocytes, together with the other clinical factors. Our results confirmed the previous findings that the factors including younger age, female, and early stage were associated with favorable prognosis for NPC patients [24]. More importantly, we found that an elevated LMR was significantly associated with better OS, DFS and DMFS and independent of other variables to predict the prognosis for NPC patients.

For the first time, we jointly considered the counts of lymphocyte and monocyte and assessed the prognostic role of LMR for clinical outcomes in NPC patients, though the association of LMR with cancer survival has been reported in classical Hodgkin's lymphoma (cHL) and diffuse large B-cell lymphoma (DLBCL) [25], [26]. We found that an elevated LMR not only had a strong correlation with better survival, including OS, DFS and DMFS, but also was an independent prognostic factor for survival in the multivariate analysis under the Cox model. These were supportive to the previous findings on cHL, where an elevated LMR had a significantly better OS and DFS [27]. Similar results were also found in DLBCL, which is another hematological malignancy [26]. These suggest that these immune cells may play similar roles in NPC and hematological malignancies. Interestingly, these two categories of tumor have been showed to share some common etiological factors. First, NPC is commonly known as an EBV related malignancy, while EBV contribution has also been implicated in HL and DLBCL [12], [13]. Secondly, HLA is consistently reported risk locus for NPC [28], [29], and it's also important locus that harbors risk gene for lymphoma [11].

Moreover, either lymphocyte count or monocyte count alone was associated with clinical outcomes in NPC patients. Our study here showed that the absolute lymphocyte count in NPC patients was significantly associated with OS and DMFS and it was an independent prognostic predictor for patients' survival. These were in consistent with the previous findings in NPC and other cancers such as ovarian and breast cancers, where the high lymphocyte count was reported as an independent favorable prognostic factor [30], [31], [32]. By contrast, pretreatment lymphopenia has been considered as an indicator for poor outcomes [27], [33]. The favorable role of lymphocytes seems biologically plausible. Lymphocytes are crucial components of host immunity that are important to destruct residual tumor cells and related micrometastases [34], [35], and infiltrating lymphocytes are able to activate an effective antitumor cellular immune response [36]. Moreover, T lymphocytes could help to drive the cancer cells towards apoptosis due to the state of chronic activation in cancer patients [37], and lead to the death of cancer cells in response to chemotherapy by presenting tumor-associated antigens to immune cells [38], [39]. NPC tumor is commonly infiltrated with T lymphocytes such as Th17 cells. Partly regulated by macrophage migration inhibitory factor (MIF), Th17 cells could produce higher levels of cytokines including TNF and IFN-**γ**, showing anti-cancer effect [40], [41]. In NPC, the expression of MIF is found in infiltrating lymphocytes including Th17 cells, and the high expression of MIF is associated with better outcomes [42].

On the other hand, the infiltrated monocytes in tumor tissue have been shown with abilities to promote tumor invasion and cell growth in lymphoma [43]. Moreover, the large number of pretreatment monocytes was associated with poor prognosis in lymphoma and other solid tumors [19], [44], [45]. Supportively, our results showed that the higher monocyte count was significantly associated with poor survival in NPC patients and it was an independent factor for prognosis prediction. In contract, a previous report didn't reveal the association of the percentage of peripheral monocyte with OS and DFS in NPC [32]. The discordance between these two studies might be partially due to the different length of follow-up, where the mean duration was 67 months here and was 41 months in the previous study. Although the elevated number of monocyte might be an unfavorable factor for cancer prognosis, the underlying mechanism between monocytes and cancer development is unclear. Monocytes could secrete various proinflammatory cytokines, such as interleukin (IL)-1, IL-6, IL-10 and TNF-á, which have been associated with shorter survival and worse prognosis in malignances [46], [47]. Moreover, monocytes are able to release monocyte chemo-attractant protein (MCP-1)-1 upon stimulation and mediate tumor-associated macrophage infiltration in solid tumors, which could produce a variety of chemokines such as TGF-â, TNF-á, IL-1 and IL-6 to promote tumorigenesis, angiogenesis and distant metastasis of malignant tumors [48], [49]. Therefore, monocytes in NPC seem to promote tumor progression, acting opposite role as lymphocytes, while further functional studies are needed to confirm these.

Although lymphocyte count or monocyte count alone could predict the survival outcomes in NPC patients, the LMR was shown to outperform better than them. Firstly, both of univariate and multivariate analysis showed that the correlation between LMR and patients' survival was much more significant than that those between lymphocyte count or monocyte count and patients' survival. Secondly, as lymphocyte count was associated with superior survival and monocyte count was correlated with inferior survival, the LMR derived from the two variables expanded the predictive value for the NPC survival, in a way to enlarge the favorable effect of lymphocytes against the unfavorable effect of monocytes in tumor progression. Lastly, the interaction between the lymphocytes and monocytes might be contributing into the performance. Previous studies demonstrated that normal human monocytes suppressed either the phytohemagglutinin (PHA) or antigen-induced lymphocytes proliferative response when the monocyte-lymphocyte ratio was increased [50]. Also, human monocytes could be stimulated to secrete large amounts of prostaglandins *in vitro*, which have been shown to inhibit mitogen-induced lymphocyte response, resulting in suppressing the anti-cancer immunity in various cancers [51], [52], [53]. Therefore, these might be the possible explanations that either an elevated lymphocyte count or depressed monocyte count was favorable prognostic predictor in NPC patients.

## Conclusions

Taken together, we firstly demonstrated that pretreatment peripheral LMR at diagnosis could function as an independent prognostic factor for patients' survival, which elevated in about 40% of NPC cases. Further, we also demonstrated that peripheral LMR was an independent biomarker for predicting clinical outcomes of NPC patients. Technically, this biomarker was directly derived from routine blood cell counts and easily applied in clinical work. In addition, we acknowledged that this finding is limited on a retrospective study in a single center, and thus further studies in either multicenter or prospective manner are awaited to validate the clinical usages of LMR as a prognostic marker for NPC.

## Supporting Information

Figure S1 **ROC curve analyses of LMR for OS (A), DFS (B), DMFS (C) and LRRFS (D).** ROC, receiver operating characteristic; A, area under the curve (AUC); LMR, lymphocyte-to-monocyte ratio.
(TIF)

Table S1 **Baseline clinical characteristics of the 1547 nasopharyngeal carcinoma patients according to lymohocyte-to-monocyte ratio.** Abbreviation: CRT  =  chemoradiotherapy; RT  =  radiotherapy; a Wilcoxon rank-sum test. b χ2 test by two-sided Pearson's exact test.
(DOC)

Table S2 **Multivariate analysis of independent prognostic factors (with lymphocyte and monocyte counts in the model; n = 1547).** Abbreviation: OS  =  Overall Survival; DFS  =  Disease-Free Survival; DMFS  =  Distant Metastasis-Free Survival; LRRFS  =  Loco-Regional Recurrence-Free Survival; CRT  =  chemoradiotherapy; RT =  radiotherapy; LY  =  lymphocyte count; MO  =  monocyte count.
(DOC)
