# Supplementary figures and images for: A Large Cohort Study Reveals the Association of Elevated Peripheral Blood Lymphocyte-to-Monocyte Ratio with Favorable Prognosis in Nasopharyngeal Carcinoma
Source: PLoS One. 2013 Dec 27;8(12):e83069. doi: 10.1371/journal.pone.0083069 (PMC3873908; doi:10.1371/journal.pone.0083069)

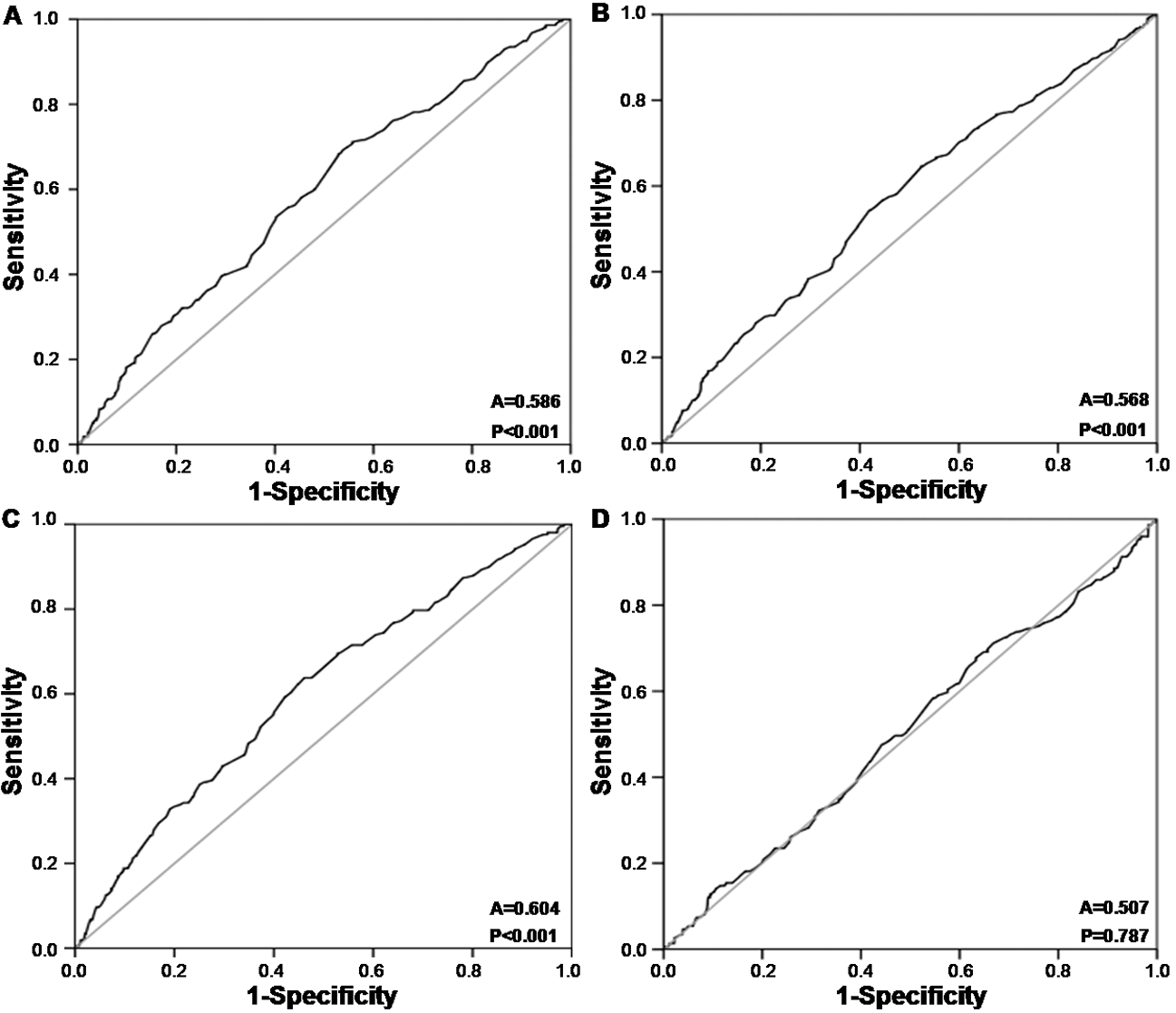

Supplement: Figure S1 — ROC curve analyses of LMR for OS (A), DFS (B), DMFS (C) and LRRFS (D). ROC, receiver operating characteristic; A, area under the curve (AUC); LMR, lymphocyte-to-monocyte ratio. (TIF) [file pone.0083069.s001.tif]
