# Supplementary material for: A Large Cohort Study Reveals the Association of Elevated Peripheral Blood Lymphocyte-to-Monocyte Ratio with Favorable Prognosis in Nasopharyngeal Carcinoma
Source: PLoS One. 2013 Dec 27;8(12):e83069. doi: 10.1371/journal.pone.0083069 (PMC3873908; doi:10.1371/journal.pone.0083069)
Supplement: Table S1 — Baseline clinical characteristics of the 1547 nasopharyngeal carcinoma patients according to lymohocyte-to-monocyte ratio. Abbreviation: CRT = chemoradiotherapy; RT = radiotherapy; a Wilcoxon rank-sum test. b χ2 test by two-sided Pearson's exact test. (DOC) [file pone.0083069.s002.doc]

| **Table S1. Baseline clinical characteristics of the 1547 nasopharyngeal carcinoma patients according to lymohocyte-to-monocyte ratio** | | | | |  |
| --- | --- | --- | --- | --- | --- |
| **characteristic** | **Overall** | **<5th percentile n=77** | **5-95th percentile n=1389** | **>5th percentile n=81** | **p-value** |
| **Median age (years)** | 51 | 53 | 52 | 55 | 0.57^a^ |
| **Gender (n,%female)** | 423/1547(27.3%) | 19/77(24.7%) | 377/1389(27.1%) | 27/81(33.3%) | 0.413^b^ |
| **T-classification (n,%)** |  |  |  |  |  |
| T1-T2 | 534 | 17 | 489 | 28 | 0.062^b^ |
| T3-T4 | 1013 | 60 | 900 | 53 |  |
| **N-classification** |  |  |  |  |  |
| N0-N1 | 846 | 45 | 758 | 43 | 0.768^b^ |
| N2-N3 | 701 | 32 | 631 | 38 |  |
| **Overall stage** |  |  |  |  |  |
| I-II | 334 | 9 | 306 | 19 | 0.091^b^ |
| III-IV | 1213 | 68 | 1083 | 62 |  |
| **Treatment** |  |  |  |  |  |
| RT | 493 | 23 | 438 | 32 | 0.303^b^ |
| CRT | 1054 | 54 | 951 | 49 |  |
| Lymphocyte Count (10**^9^/L)(mean)** | 2.13 | 1.41 | 2.143 | 2.512 | <0.001^a^ |
| Monocyte Count (10**^9^/L)(mean)** | 0.46 | 0.818 | 0.456 | 0.193 | <0.001^a^ |

Abbreviation: CRT=chemoradiotherapy; RT= radiotherapy;

aWilcoxon rank-sum test

bχ2 test by two-sided Pearson’s exact test
