# Supplementary material for: A Large Cohort Study Reveals the Association of Elevated Peripheral Blood Lymphocyte-to-Monocyte Ratio with Favorable Prognosis in Nasopharyngeal Carcinoma
Source: PLoS One. 2013 Dec 27;8(12):e83069. doi: 10.1371/journal.pone.0083069 (PMC3873908; doi:10.1371/journal.pone.0083069)
Supplement: Table S2 — Multivariate analysis of independent prognostic factors (with lymphocyte and monocyte counts in the model; n = 1547). Abbreviation: OS = Overall Survival; DFS = Disease-Free Survival; DMFS = Distant Metastasis-Free Survival; LRRFS = Loco-Regional Recurrence-Free Survival; CRT = chemoradiotherapy; RT = radiotherapy; LY = lymphocyte count; MO = monocyte count. (DOC) [file pone.0083069.s003.doc]

| **Table S 2. Multivariate analysis of independent prognostic factors (with lymphocyte and monocyte counts in the model; n=1547)** | | | | | | | | |
| --- | --- | --- | --- | --- | --- | --- | --- | --- |
| **Variable** | **OS** | | **DFS** | | **DMFS** | | **LRRFS** | |
|  | **HR (95% CI)** | ***P* value** | **HR (95% CI)** | ***P* value** | **HR (95% CI)** | ***P* value** | **HR (95% CI)** | ***P* value** |
| **Gender (male vs. female)** | 1.986(1.374-2.870) | ＜0.001 | 1.727(1.310-2.276) | ＜0.001 | 1.765(1.230-2.532) | 0.002 | 1.612(1.074-2.420) | 0.021 |
| **Age (≥ vs.＜51 years)** | 1.891(1.418-2.522) | ＜0.001 | 1.169(0.941-1.452) | 0.157 | 1.410(1.064-1.868) | 0.017 | 0.948(0.686-1.311) | 0.747 |
| **T status (T3-T4 vs. T1-T2)** | 1.690(1.079-2.648) | 0.022 | 1.444(1.017-2.050) | 0.04 | 1.534(0.992-2.371) | 0.054 | 1.185(0.689-2.039) | 0.54 |
| **N status (N2-N3 vs. N0-N1)** | 1.438(1.059-1.952) | 0.02 | 1.391(1.079-1.794) | 0.011 | 1.645(1.192-2.269) | 0.002 | 1.047(0.706-1.552) | 0.82 |
| **Overall stage (III-IV vs. I-II)** | 1.600(0.804-3182) | 0.181 | 0.991(0.608-1.618) | 0.973 | 1.166(0.600-2.266) | 0.649 | 1.054(0.518-2.145) | 0.884 |
| **Treatment (CRT vs. RT)** | 1.151(0.820-1.616) | 0.417 | 1.175(0.900-1.536) | 0.236 | 1.347(0.941-1.930) | 0.104 | 0.945(0.645-1.383) | 0.77 |
| **Lymphocyte Count (10^9^/L)  (≥ vs.＜2.145)** | 0.586(0.441-0.779) | ＜0.001 | 0.742(0.595-0.925) | 0.008 | 0.653(0.491-0.869) | 0.003 | 0.918(0.661-1.276) | 0.612 |
| **Monocyte Count (10^9^/L)  (≥ vs.＜0.475)** | 1.426(1.086-1.874) | 0.011 | 1.318(1.061-1.638) | 0.013 | 1.533(1.160-2.027) | 0.003 | 1.034(0.745-1.435) | 0.841 |
| Abbreviation: OS=Overall Survival; DFS=Disease-Free Survival; DMFS=Distant Metastasis-Free Survival; LRRFS=Loco-Regional Recurrence-Free Survival; CRT=chemoradiotherapy; RT= radiotherapy; LY=lymphocyte count; MO=monocyte count | | | | | | | | |
